# Whole Genome Identification and Integrated Analysis of Long Non-Coding RNAs Responding ABA-Mediated Drought Stress in *Panax ginseng* C.A. Meyer

**DOI:** 10.3390/cimb47010005

**Published:** 2024-12-25

**Authors:** Peng Chen, Cheng Chang, Lingyao Kong

**Affiliations:** College of Life Sciences, Qingdao University, Qingdao 266071, China; chenpeng1@qdu.edu.cn (P.C.); cc@qdu.edu.cn (C.C.)

**Keywords:** long non-coding RNA, high-throughput sequencing, *Panax ginseng*, ABA, drought stress

## Abstract

*Panax ginseng* C.A. Meyer is a perennial herb that is used worldwide for a number of medical purposes. Long non-coding RNAs (lncRNAs) play a crucial role in diverse biological processes but still remain poorly understood in ginseng, which has limited the application of molecular breeding in this plant. In this study, we identified 17,478 lncRNAs and 3106 novel mRNAs from ginseng by high-throughput illumine sequencing. 50 and 257 differentially expressed genes (DEGs) and DE lncRNAs (DELs) were detected under drought + ABA vs. drought conditions, respectively. The DEGs and DELs target genes main enrichment is focused on the “biosynthesis of secondary metabolites”, “starch and sucrose metabolism”, and “carbon metabolism” pathways under drought + ABA vs. drought conditions according to KEGG pathway enrichment analysis, suggesting that these secondary metabolites biosynthesis pathways might be crucial for ABA-mediated drought stress response in ginseng. Together, we identified drought stress response lncRNAs in ginseng for the first time and found that the target genes of these lncRNAs mainly regulate the biosynthesis of secondary metabolites pathway to response to drought stress. These findings also open up a new visual for molecular breeding in ginseng.

## 1. Introduction

Drought is one of the primary abiotic stress factors that seriously restricts plant development and regional distribution, as well as lowers crop yields. In order to mitigate the detrimental effects of water scarcity on plant growth and reproduction, plants primarily adapt to drought stress by altering their physiology, metabolism, and development [1]. Abscisic acid (ABA), a phytohormone, is the principal signaling molecule for plants to respond to drought stress. When plants are subjected to drought stress, ABA content will increase rapidly by the biosynthesis pathway. Meanwhile, osmoregulatory compounds such as soluble sugar and proline also accumulate in plants to lessen the damage caused by drought stress [2]. Fascinatingly, the exogenous application of ABA can alleviate the damage caused by drought when plants are subjected to sustained drought stress [2]. Many distinct studies have revealed the signaling regulating pathway of ABA response to drought stress, including ABA receptors (PYRABACTIN RESISTANCE1/PYR1-LIKE/REGULATORY COMPONENTS OF ABA RECEPTORS, PYR/PYL/RCAR), type 2C protein phosphatases (PP2Cs), protein kinases (SUCROSE NONFERMENTING1-RELATED PROTEIN KINASES SUBFAMILY2, SnRK2s) [3,4,5] and downstream components, such as transcriptional factors, ABA and stress-response genes [5,6]. In addition, SnRK2s, a type of protein kinases, also phosphorylate and activate NADPH oxidases, SLAC1 (SLOW ANION CHANNEL1, SLAC1), and AQPs (aquaporins, AQPs) [7,8,9]. These signaling molecules and channel proteins directly induce stomatal closure to prevent water loss in drought.

Furthermore, numerous potential genes/QTLs (like *qPH10.1*, *qEPN6.1*, qPL9.1, and so on) and various molecular signaling cascades regulatory networks (such as MAPK cascade) were uncovered to associate with drought resistance with the advances of molecular biology in plant [10,11]. Subsequently, a large amount of drought-responsive miRNAs and their candidate target genes were discovered in various cereal crops including barley and wheat, revealing the molecular mechanisms of drought stress adaptation [12]. Similarly, drought-responsive lncRNAs were found in *Arabidopsis thaliana*, rice, maize, peanut, and other plant species [13], indicating that lncRNAs play a role in response to drought tolerance. Nonetheless, the function of lncRNAs in medicinal ginseng drought resistance is still in its infancy.

Known as the king of all herbs, *Panax Ginseng* is a type of perennial herbaceous plant of the Araliaceae family that has been used for medicinal purposes for thousands of years [14]. It is commonly known that ginsenosides are the main active ingredients in ginseng, which has remarkable therapeutic effects on various diseases, including cancer [15,16], diabetes mellitus [17,18], cardiovascular disorders [19], neurological disorders [20,21], even cervical spondylotic myelopathy [20]. Furthermore, ginsenosides, serving as functional ingredients, are widely utilized in many health products, functional foods, and cosmetics [22]. Ginsenosides are mostly produced in the roots, but they are also deposited in other tissues such as leaves, stems, flower buds, and berries [14]. However, the growth of ginseng and the concentration of ginsenosides are influenced by various environmental challenges, including biotic and abiotic stressors [23]. Drought stress is one of the most serious abiotic stressors during the ginseng growth period [24,25]. Kong et al. reported that phytohormone abscisic acid (ABA) enhanced the drought resistance, and increased ginsenoside accumulation in both drought and drought + ABA treatment in *Panax ginseng* [2]. However, more detailed regulatory mechanisms still remain poorly studied in ginseng response to drought and drought + ABA.

Long non-coding RNAs (lncRNAs), accounting for the vast majority of non-coding RNAs, are a type of non-coding RNAs with a length of ≥200 nucleotides [26]. Generally, lncRNAs have low sequence conservation and strong tissue and spatiotemporal expression specificity [27]. Based on the genomic localization relative to protein-coding genes, lncRNAs are classified into three major categories: long intergenic non-coding RNAs (lincRNAs), long intron non-coding RNAs (lncRNAs), and long non-coding natural antisense transcripts (NATs) [28]. Numerous studies carried out in recent years have demonstrated the multiple biological functions of lncRNAs in plant growth and development, such as seedling photomorphogenesis, blooming, fruit ripening, reproduction, and environmental stress response [29,30,31,32]. For the past decades, extensive lncRNAs were identified to response abiotic stress in diverse plant species, including *Arabidopsis thaliana* [31,33], *Zea mays* [34,35,36], *Oryza sativa* [37,38], and *Gossypium hirsutum* [39]. Zhang et al. identified 664 high-confidence drought-responsive lncRNAs from 1724 potential non-coding RNAs in maize (*Zea mays*) [35]. Chung et al. identified 98 lncRNAs that were regulated by drought stress in rice [38]. And 19 lncRNAs responded to simulated drought stress in foxtail millet (*Setariaitalica*) [40]. Nowadays, more studies are focused on comprehensively explaining the biological functions and molecular mechanisms of lncRNAs response to abiotic stress. For instance, Chen et al. reported that overexpression of *lncRNA 77580* enhanced the drought tolerance of soybean [41]. In rice, lncRNA *TCONS_00021861* positively regulates YUCCA7 expression through sponge *miR528-3p*, activating the indoleacetic acid (IAA) biosynthetic pathway to resist drought stress [42]. In Arabidopsis, a novel lncRNA *DRIR* (*DROUGHT INDUCED lncRNA*) was induced expression by drought and salt stress, thereby enhancing drought and salt stress tolerance. Subsequent research showed that *DRIR* regulates the expression of stress response genes involved in the ABA response and water transport [31]. Zhang et al. revealed that lncRNA *DANA2* interacts with ERF84 (an AP2/ERF transcription factor) and then positively regulates the drought tolerance by modulate the transcription of JMJ29 in Arabidopsis [43]. Another research by the same group demonstrated that drought-induced long intergenic non-coding RNA lncRNA *DANA1* interacted with the novel chromatin-related factor DIP1 (DANA1-INTERACTING PROTEIN 1). *DANA1*-DIP1 complex increased histone deacetylase HDA9 activity and decreased CYP707A1 and CYP707A2 acetylation, and then positively regulated drought response [33]. However, the lncRNAs that respond to drought stress in ginseng have not been studied.

In order to investigate the roles of lncRNAs in response to ABA-modulated drought stress in ginseng, we performed transcriptome sequencing and analysis in ginseng under various drought and drought + ABA treatments in this study. We identified 17,478 lncRNAs and 3106 novel mRNAs. The differentially expressed lncRNA were screened, and their target genes were predicted. GO annotation and KEGG pathway analysis were employed for target genes classification, function analysis, and biological pathway analysis. Finally, our findings will be helpful for further understanding the molecular mechanism of ginseng response to drought stress and provide a perspective on the molecular breeding in ginseng.

## 2. Materials and Methods

### 2.1. Plant Materials and Culture Conditions

In this study, 3 years old ginseng (*Panax ginseng* C.A. Meyer) seedlings were employed, which were obtained from Tonghua ginseng industrial farm, in Jilin, China. The dormant ginseng roots with spore were purchased and soaked in 200 ppm gibberellin acid for 4 h to disrupt dormancy. Then, these ginseng roots were planted in the 1:1 mixture of organic soil and vermiculite, then cultured in the greenhouse with 50 μM m^−2^ s^−1^ light at 22 °C and 16 h light/8 h dark for 4 weeks.

### 2.2. Drought and Drought + ABA Treatment

For drought treatment, 3-year-old ginseng seedlings were cultivated in normal conditions for 4 weeks, and then water supply was stopped for 35 days as drought treatment. Meanwhile, another group was sprayed with 15 μM ABA (Sigma-Aldrich, St. Louis, MO, USA) every two days after the water supply was stopped, which serves as drought + ABA treatment. The control group was watered regularly and without ABA spraying. After 35 days, when the leaves were visibly wilting, root materials were collected. Each treatment condition collects three roots samples as three biological replicates, and each replication sample contains five ginseng root materials. After collection, the samples were promptly snap-frozen with liquid nitrogen and stored at −80 °C for RNA extraction.

### 2.3. RNA Extraction and Library Preparation

RNA isolation was conducted as described by Lei et al. [44]. The total RNA of each sample was isolated by RNeasy Plant Mini Kit (QIAGEN, CAT.NO. 74904, Hilden, Germany). Then, ribosomal RNAs were removed by Illumina Ribo-Zero rRNA Removal Kit to prepare a specific library. 3 μg of RNA (per sample) were employed and NEBNext ^®^ The UltraTM RNA Library Prep Kit for Illumina ^®^ (NEB, #E3330, Ipswich, MA, USA) was used to generate sequencing libraries according to the kit instructions. Briefly, mRNA was purified from total RNA by poly-T oligo-attached magnetic beads. Fragmentation was performed using divalent cations under elevated temperature in NEB Next First Strand Synthesis Reaction Buffer. First-strand cDNA was synthesized via random hexamer primer and M-MuLV Reverse Transcriptase (RNase H^−^). Then the second strand cDNA synthesis was carried out by DNA Polymerase I and RNase H. Remaining overhangs were converted into blunt ends by exonuclease/polymerase activities. After the 3′ end of the DNA fragment is adenylated, connect it to a NEB Next adapter with a hairpin loop to prepare for hybridization. In order to select cDNA fragments with a length of 150–200 bp, the library fragments were purified using the AMPure XP system (Beckman Coulter, Beverly, MA, USA). Then 3 μL USER Enzyme (NEB,#M5509, USA) was employed with size-selected, adaptor-ligated cDNA at 37 °C for 15 min followed by 5 min at 95 °C before PCR. Then PCR was performed with Phusion High-Fidelity DNA polymerase, Universal PCR primers, and Index (X) Primer. At last, PCR products were purified by AMPure XP system (Beckman Coulter, A63880, Brea, CA, USA) and library quality was assessed on the Agilent Bioanalyzer 2100 system.

### 2.4. Differential Expression Analysis

Differential expression analysis of two conditions/groups (three biological replicates per condition) was performed by the DESeq2 R package (1.10.1). DESeq2 provides statistical routines for determining differential expression in digital gene expression data using a model based on the negative binomial distribution. The resulting *p*-values were adjusted by the Benjamini and Hochberg’s approach for controlling the false discovery rate. Genes/lncRNAs with the false discovery rate adjusted absolute fold-change ≥ 2 and *p*-value < 0.05 were assigned as differentially expressed genes/lncRNAs.

### 2.5. Gene Ontology and Kyoto Encyclopedia of Genes and Genomes Enrichment Analysis

Gene Ontology (GO) enrichment analysis of differentially expressed genes was carried out by the clusterProfiler R package, in which gene length bias was corrected. GO terms with corrected *p*-value < 0.05 were considered significantly enriched by differentially expressed genes (DEG) and differential expression lncRNAs (DE lncRNAs).

KEGG is a database resource for understanding high-level functions and utilities of the biological system, such as the cell, the organism, and the ecosystem, from molecular-level information, especially large-scale molecular datasets generated by genome sequencing and other high-throughput experimental technologies (http://www.genome.jp/kegg/) (accessed on 27 August 2019). We used clusterProfiler R package to test the statistical enrichment of DEGs and DE lncRNAs target genes in KEGG pathways.

## 3. Results

### 3.1. High-Throughput Sequencing and Analysis Workflow Developing

**Figure 1:**
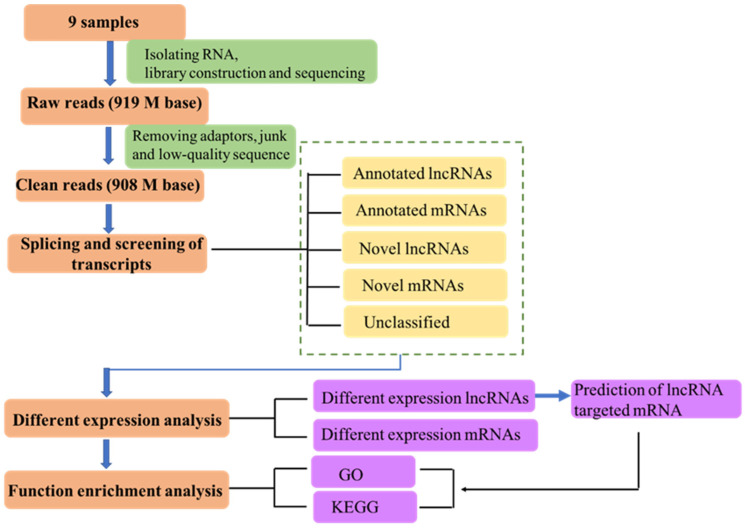
The RNA sequencing and bioinformatic analysis workflow in this study. The orange box shows the process of sequencing and bioinformatic analysis. The green box illustrates the data source and criteria quality control. LncRNAs and mRNAs identification were showed in the yellow box. Differential expression analysis and function enrichment analysis are displayed in the purple box.

Our previous work demonstrated that ABA plays a vital role in alleviating the physiological phenotype caused by drought stress [2]. In order to identify lncRNAs involved in ABA-mediated drought stress in ginseng, we treated three-year-old ginseng seedlings with drought and drought + ABA. After 35 days of treatment, RNA was collected from the roots for library construction and sequencing. A total of 9 samples were obtained under control (CK), drought, and drought + ABA treatment with three biological repetitions, respectively. To make the sequencing and analytic process more obvious, we summarized our approach in Figure 1 (Figure 1).

A total of 919 million raw reads were obtained from the 9 transcriptome libraries by high-throughput illumine sequencing. And 908 million clean reads were generated after removing adaptors, junk, and low-quality sequences (Table 1). Then, all the clean reads were mapped to ginseng reference genome (http://ginsengdb.snu.ac.kr/data) (accessed on 27 August 2019) by HISAT2 software (2.1.0), and more than 94% of the reads were aligned successfully.

**Figure 2:**
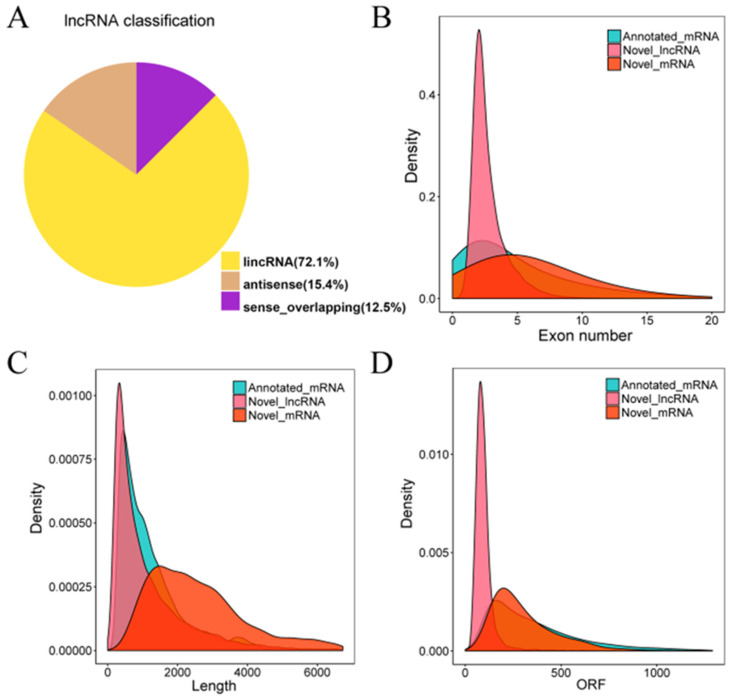
A comprehensive analysis of lncRNAs and mRNAs in ginseng roots. (**A**) Pie diagram shows the counts of different kind of lncRNAs. (**B**–**D**) Comparison of exon number, transcript length and ORF length between lncRNAs and mRNAs.

Through sequencing, 17,478 lncRNAs (length ≥ 200 bp, ORF cover ≤ 0.5, TMP > 1, potential coding scores ≤ 0.5, coding potential < 0) were identified. These lncRNAs were characterized based on the relative locations to the nearest protein-coding genes. The majority of the lncRNAs (72.1%) were located in the intergenic regions (lincRNA), 15.4% of antisense lncRNAs, and 12.5% of sense-overlapping lncRNAs (Figure 2A). Compared with mRNA, these lncRNAs generally have fewer exons (Figure 2B), shorter sequence lengths, and shorter Open Reading Frames (ORFs) (Figure 2C,D), which confirmed that the lncRNAs we identified were consistent with previous reports and adhere to the general characteristics of lncRNAs [45]. Then, these identified lncRNAs were subjected to follow-up analyze.

### 3.2. Identification of DEGs

**Figure 3:**
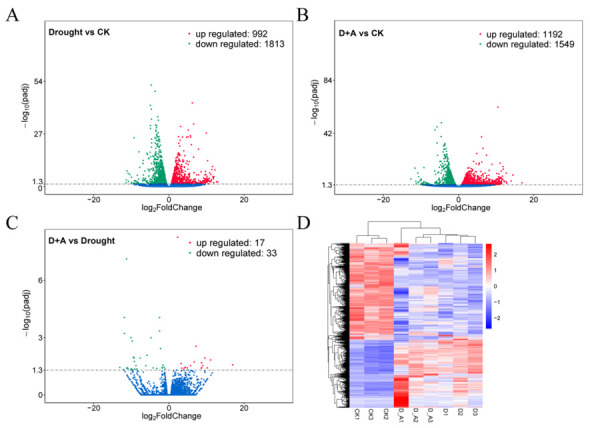
Differentially expressed genes (DEGs) at the ginseng root tissue under different treatment conditions. D + A, represented drought + ABA treatment. (**A**) Volcano plots of DEGs in drought vs. CK. (**B**) Volcano plots of DEGs in D + A vs. CK. (**C**) Volcano plots of DEGs in D + A vs. drought. Upregulated, down-regulated, and non-differentially expressed genes are represented by red, green, and blue dots, respectively. (**D**) Heatmap and cluster analysis of the expression level of DEGs. CK1, CK2, CK3, D1, D2, D3, D + A1, D + A2, and D + A3 represent three repetitions of control, drought, and drought +ABA treatment, respectively.

To investigate the gene expression profile of ginseng roots under drought stress and drought + ABA treatment, the expression levels were analyzed based on the FPKM (Fragments Per Kilobase of transcript sequence per Millions base pairs sequenced) values. Compared with the control (CK), 2805 differentially expressed genes (DEGs) were identified in drought treatment, including 992 up-regulated and 1813 down-regulated genes, respectively (Figure 3A and Table S1) (Table S1 showed all the DEGs in Figure 3A). In the condition of drought + ABA, 2741 DEGs were identified compared to CK, including 1192 up-regulated and 1549 down-regulated genes, respectively (Figure 3B and Table S2) (Table S2 showed all the DEGs in Figure 3B). However, compared drought and drought + ABA, only 50 DEGs were identified, including 17 up-regulated and 33 down-regulated genes, respectively (Figure 3C and Table S3) (Table S3 showed all the DEGs in Figure 3C). Correlation analysis showed that different samples at the same treatment conditions had a high consistency and were clustered together (Figure 3D). These results indicated significant differences in gene expression under drought and drought + ABA compared to normal conditions, and these DEGs might be essential for ABA-mediated drought stress response.

### 3.3. Functional Enrichment of Drought Resistance-Related DEGs

**Figure 4:**
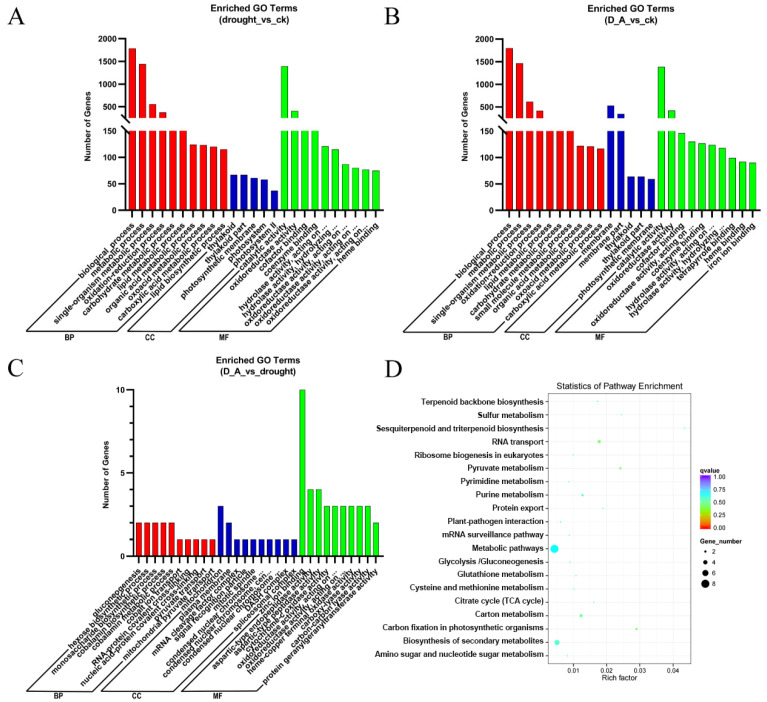
Comparison of Gene Ontology (GO) classification and Kyoto Encyclopedia of Genes and Genomes (KEGG) pathway enrichment analysis of differentially expressed genes (DEGs). (**A**–**C**) GO analysis of DEGs in drought vs. control, drought + ABA vs. control and drought + ABA vs. drought, respectively. (**D**) KEGG pathway enrichment analysis of DEGs in drought + ABA vs. drought. The size of the dot indicates the number of DEGs in the corresponding pathway. BP, CC, and MF represent biological processes (BP), cellular components (CC), and molecular functions (MF), respectively.

GO and KEGG enrichment analysis were employed to explore the biological function of these DEGs. For GO enrichment analysis, we conducted analysis in three aspects: biological processes (BP), cellular components (CC), and molecular functions (MF). For each section, we only display the top 10, 5, and 10 terms as the most significantly enriched GO terms in BP, CC, and MF, respectively. The results uncovered that under the conditions of drought vs. CK and drought + ABA vs. CK, the differentially expressed genes were mainly concentrated in biological processes and molecular functions. Among them, more genes were enriched in the metabolic process (GO:0008152), biological process (GO:0008150), and catalytic activity (GO:003824) terms (Figure 4A,B; Tables S4 and S5) (Tables S4 and S5 showed GO enrichment results in Figure 4A,B). However, the genes enriched during drought + ABA vs. drought processes mainly participate in the regulation of molecular functions such as aspartic-type peptidase activity (GO:0070001), aspartic-type endopeptidase activity (GO:0004190) and zinc ion binding (GO:0008270) (Figure 4C and Table S6) (Table S6 showed GO enrichment results in Figure 4C), suggesting that ABA mainly induces these molecular functional related genes expression during the process of alleviating damage caused by drought stress. In addition, under drought + ABA vs. drought conditions, Kyoto Encyclopedia of Genes and Genomes (KEGG) enrichment analysis indicated that “metabolic pathways”, “biosynthesis of secondary metabolites” and “carbon metabolism” were significantly enriched (Figure 4D), suggesting that these pathways play an important role in ABA-mediated drought stress response.

### 3.4. Identifications of Differentially Expressed lncRNAs (DELs)

**Figure 5:**
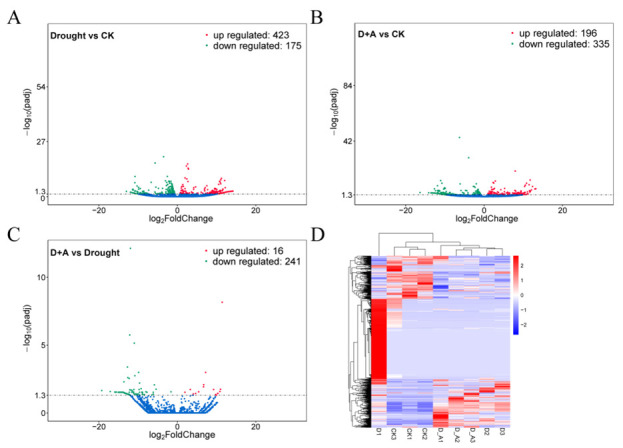
Differentially expressed lncRNAs (DELs) at the ginseng root tissue under different treatment conditions. D + A, represented drought + ABA treatment. (**A**) Volcano plots of DELs in drought vs. CK. (**B**) Volcano plots of DELs in D + A vs. CK. (**C**) Volcano plots of DELs in D + A vs. drought. Up-regulated, down-regulated, and non-differentially expressed lncRNAs are represented by red, green, and blue dots, respectively. (**D**) Heatmap and cluster analysis of the expression level of DELs. CK1, CK2, CK3, D1, D2, D3, D + A1, D + A2, and D + A3 represent three repetitions of control, drought, and drought +ABA treatment, respectively.

To identify differentially expressed lncRNAs under drought and drought + ABA, lncRNAs with expression changes of at least 2.0-fold and *p*-value ≤ 0.05 were considered differentially expressed. A total of 598 DELs were identified under drought conditions compared to CK, including 423 up-regulated and 175 down-regulated DELs, respectively (Figure 5A and Table S7) (Table S7 showed different expressional lncRNAs in Figure 5A). Compared with CK, 531 DELs were identified under drought + ABA, including 196 up-regulated and 335 down-regulated DELs, respectively (Figure 5B and Table S8) (Table S8 showed different expressional lncRNAs in Figure 5B), However, compared drought and drought + ABA, only 257 DELs were identified, including 16 up-regulated and 241 down-regulated DELs, respectively (Figure 5C and Table S9) (Table S9 showed different expressional lncRNAs in Figure 5C). These findings showed that these DELs involved in ABA-mediated drought stress and improved the drought tolerance in ginseng root. Moreover, hierarchy analysis showed that different samples at the same treatment conditions clustered together (Figure 5D).

### 3.5. Prediction and Enrichment Analysis of lncRNA Targets Genes Responding to Drought Stress

To investigate the biological functions of the identified DE lncRNAs in ginseng root, two methods were employed to predict the target genes of these DE lncRNAs. One is co-location analysis, which predicts cis-target genes based on the positional relationship between lncRNAs and mRNAs, with a screening range of up to 100 kb. Results showed that 15,228 lncRNAs-21,945 mRNAs pairs were identified through co-location analysis (Table S10) (Table S10 showed predicted lncRNAs-mRNAs co-location result).

**Figure 6:**
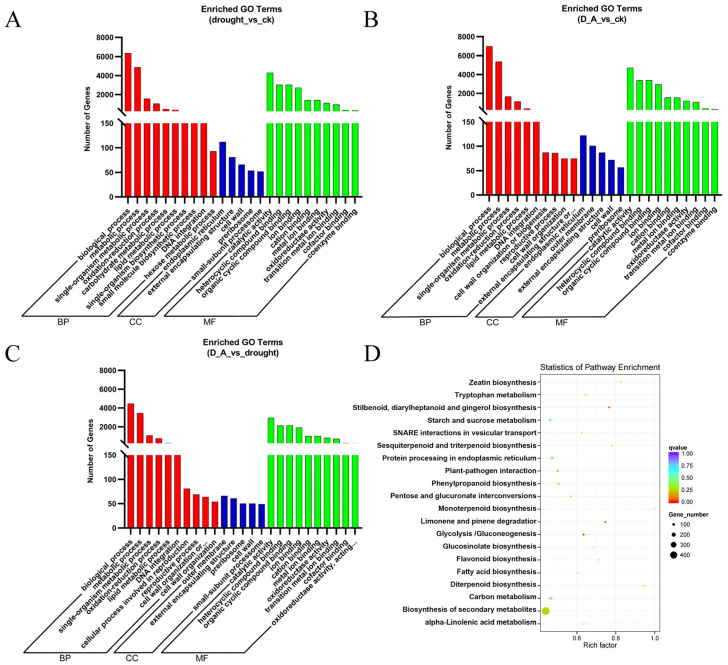
Comparison of Gene Ontology (GO) classification and Kyoto Encyclopedia of Genes and Genomes (KEGG) pathway enrichment analysis of the target genes of differentially expressed lnCRNAs (DELs). (**A**–**C**) GO analysis of these target genes in drought vs. control, drought + ABA vs. control and drought + ABA vs. drought, respectively. (**D**) KEGG pathway enrichment analysis of target genes of lncRNAs in drought + ABA vs. drought treatment. The size of the dot indicates the number of target genes in the corresponding pathway. BP, CC, and MF represent biological processes (BP), cellular components (CC), and molecular functions (MF), respectively.

Another approach is expression-related target gene analysis (co-expression analysis), which predicts target genes based on the expression correlation between lncRNAs and mRNAs. 1167 lncRNAs-21,863 mRNAs pairs were identified through co-expression analysis (Table S11) (Table S11 showed predicted lncRNAs-mRNAs co-expression results). To understand the function of these lncRNAs, we carried out GO term enrichment and KEGG pathway analysis of the target genes of lncRNAs by co-expression analysis. All the predicted target genes were divided into 3 main categories: biological process (BP), cellular component (CC), and molecular function (MF). For each category, we only display the top 10, 5, and 10 terms as the significantly enriched GO terms in BP, CC, and MF, respectively. The results revealed that under the different conditions of drought, drought + ABA, and CK, the differential target genes were concentrated in the same category. In the biological processes, the most significantly enriched GO terms were biological process (GO:0008150), metabolic process (GO: 0008152), single-organism metabolic process (GO: 0044699), and oxidation-reduction process (GO: 0055114). With the cellular component category, the most significantly enriched GO terms were endoplasmic reticulum (GO: 005783), outer membrane (GO: 0019867), and external encapsulating structure (GO: 0030312). In the molecular function category, the most significantly enriched GO terms included catalytic activity (GO: 0003824) and heterocyclic compound binding (GO: 1901363) (Figure 6A–C; Tables S12–S14). In addition, KEGG pathway analysis was employed to further understand the functions of these lncRNAs target genes. All these target genes were focused on 20 different pathways by KEGG analysis. The bubble chart displays the different KEGG pathways with different target numbers. The most significant pathway including “biosynthesis of secondary metabolites”, “starch and sucrose metabolism”, and “protein processing in the endoplasmic reticulum”. These results strongly suggested that lncRNAs, serving as a type of regulator, are essential to these pathways and make an important contribution to ABA alleviating drought stress damage in ginseng (Figure 6D).

## 4. Discussion

### 4.1. LncRNAs Play a Significant Role in ABA-Mediated Drought Stress Response

Our previous studies indicated that drought stress could induce the biosynthesis of ginsenosides, the most important medicinal ingredient owing to multifaceted pharmacological properties in ginseng. More interestingly, phytohormone abscisic acid (ABA) may improve the ginsenosides synthesis and lessen the negative effects in ginseng when subjected to drought stress [2]. However, the detailed drought-responsive mechanisms in ginseng remain unknown. LncRNAs are a novel class of molecules that play important roles in various biological processes, including developmental regulation and stress response. Yet only a few lncRNAs have been proven to involve drought response in plants. To uncover the drought-responsive lncRNAs and the mechanisms underlying drought tolerance in ginseng, we first identified lncRNAs from ginseng roots with drought and drought + ABA treatments by whole transcriptome sequencing. After a strict bioinformatic analysis, a total of 1386 high-confidence lncRNAs were identified to response drought stress in ginseng. Compared to protein-coding genes, these lncRNAs had fewer exons in structures, shorter sequence lengths, and shorter ORFs [46,47]. Then we carried out the transcriptomic analysis and identified 598, 531, and 257 lncRNAs in drought vs. CK, drought + ABA vs. CK, and drought vs. drought + ABA, respectively. Most of the drought-responsive lncRNAs showed differential expressions at different treatments, especially in drought + ABA, which may explain why ABA alleviates the physiological damage phenotype when subjected to drought stress in ginseng.

Unfortunately, few studies focused on the mechanism of lncRNA in ginseng. Bian et al. reported that giant lncRNAs were found in ginseng rusty root symptom tissue. These lncRNAs involved in the homeostatic process and fatty acid-related regulation process [45]. Our results are basically consistent with previous findings, suggested that lncRNAs play a broad role in plants response to biotic and abiotic stress. The precise molecular mechanisms need to be investigated in future.

### 4.2. “Biosynthesis of Secondary Metabolites”, “Starch and Sucrose Metabolism” Pathways Play an Important Role in ABA-Mediated Drought Tolerance

At the transcription level, functional gene expression was affected by various factors, including TFs, miRNAs, and lncRNAs. LncRNAs, serving as a transcription regulatory factor, directly or indirectly regulate the expression of functional genes [46,47,48]. In this study, we identified 1167 lncRNA-21,863 mRNA pairs by co-expression analysis. To further understand the function of these DELs under drought stress and drought + ABA treatment, we performed the KEGG pathway and GO term enrichment analysis for the target genes of these DELs (Figure 6). Some drought stress-responsive pathways and GO terms, including “biosynthesis of secondary metabolites”, “starch and sucrose metabolism” pathway, and “catalytic activity”, “biological process” terms were significantly enriched with the target genes.

Similar studies have been reported in Arabidopsis, rice, and medicinal plants licorice and *Artemisia. Argyi*. In Arabidopsis, 5 flavonols, 5 anthocyanins, proline, raffinose, and galactinol were dramatically accumulated during drought stress [49]. In rice, under drought stress, 49 metabolites and 80 metabolites were identified in drought-susceptible genotype IR64 and drought-tolerant genotype Azucena, respectively. Most of these metabolites are involved in secondary metabolism, sugar alcohol metabolism, and amino acid metabolism, of which allantoin, glucose, galactaric acid, and gluconic acid showed a significant positive correlation with drought tolerance [50]. Likewise, in spikelets, ABA mediates the effect of post-anthesis soil drying on grain filling by regulating the key enzymes in sucrose-to-starch conversion [51]. In medicinal plants licorice and *Artemisia. Argyi*, ABA stimulates the biosynthesis of multiple secondary metabolites including phenylpropanoids, flavonoids, triterpenoids, and alkaloids [52,53]. These findings are consistent with the results of this study.

In addition, it is a well-known concept that drought stress is a typical environmental feature that contribute to the production of high-quality medicinal plants. Generally, drought could lead to increased secondary metabolite accumulation, such as carotenoids, lutein, and zeaxanthin [54,55]. For example, in extreme drought conditions, 15-cis-phytoene/all-trans-phytoene synthases (PSYs) (a rate-limiting enzyme in carotenoids biosynthesis) genes and the genes involved in the biosynthesis of lutein, zeaxanthin, and astaxanthin were upregulated in *P. kingianum* tubers, suggesting that drought increased accumulation of these secondary metabolites productions [56]. In rice and tea plants, salt and drought treatments also lead to upregulation expression of *OsPSY3*, *CsPSY1*, *CsPSY2*, and *CsPSY1* [57,58]. As an antioxidant, carotenoids may detoxify reactive oxygen species and enhance plant drought tolerance. These studies are consistent with our findings that secondary metabolites biosynthesis pathways were enriched in KEGG analysis in drought and drought + ABA treatment. Accordingly, it was speculated that the higher synthesis of secondary metabolites, such as carotenoid, is a manifestation of plant response to drought stress. Similar researches were reported in African eggplants, alpine plants, beans, and olive trees [59,60,61,62].

For starch and sucrose metabolism, in early tuber crops, like potatoes, studies uncovered that drought stress impairs the morphological, physiological and biochemical traits and reduces the carbon portioning and starch accumulation in potatoes [63,64]. Therefore, it is speculated that under abiotic stresses, including drought, salt, and heat, the energy changes in plants are in dynamic equilibrium. On the one hand, plants remobilize available starch to release energy to maintain survival [65], such as in *P. kingianum*, the genes coding starch and alpha-amylase synthase were upregulated under drought treatment [56]. On the other hand, sucrose synthases-related genes upregulated expression suggesting the increasing sucrose synthesis to combat drought damage in drought conditions. For example, the prolonged drought stress induced the accumulation of sucrose, proline, fructose, mannose, and malic acid in drought-tolerant wheat genotype JD17 [66]. In addition, there is another strategy to resist water stress, which is to reduce sucrose phosphate synthase expression. By this strategy, the synthesis of sucrose obviously reduced under water stress in potato growing tuber [67]. In summary, the dynamic expression changes of key genes in starch and sucrose biosynthesis pathways under drought conditions indicate that these energy substances play an important role in regulating plant drought tolerance. This is also consistent with our results that under drought and drought + ABA conditions, the active metabolic changes of starch and sucrose endow with the ability to resist the damage of drought stress in ginseng.

Interestingly, these secondary metabolites not only respond to drought stress, but also to salinity, cold, high temperature, and metal toxicity. Flavonoids and phenolic compounds are common secondary metabolites that play crucial physiological roles in abiotic stress tolerance in plants [68]. Flavonoids serve as an alleviator of oxidative and abiotic stress damage, which have the potential to scavenge reactive oxygen species (ROS) [49]. Phenolic acids are involved in plant growth and development, including photosynthesis, protein synthesis, nutrient uptake, and cytoskeleton and structure formation [69]. Increasing accumulation of these secondary metabolites under abiotic stress conditions will help the plant to adapt to environmental challenges. Therefore, there must be extensive cross-talking between various pathways and molecular mechanisms (including ROS scavenging, photosynthesis change, and so on) in plant response to abiotic stress, which works in cooperation to resist environmental constraints.

Drought tolerance research on ginseng is in its infancy. Zhou et al. identified 10,839 unigenes that could map to 91 KEGG pathways under drought and salt stress in *Panax japonicus* Meyer. Further analysis predicted that sesquiterpene and triterpene metabolic pathways, including 4 genes, involved in ginsenoside biosynthesis [70]. However, the detailed molecular mechanisms are still unclear. Advancing of transcriptome sequencing, scientists begin to focus on the important molecules that could respond to drought stress. For example, 22 PgWRKYs and 5 PgbZIPs were identified to respond to drought stress by transcriptome data analysis [71]. It is believed that the molecular mechanism of ginseng drought response will be clear with the advancement of molecular biology technology.

### 4.3. The Function of ABA in Improving Drought Tolerance in Plants

Plants have developed mechanisms to adapt and protect themselves against biotic and abiotic stress in evolution. Abscisic acid (ABA) is one of the most important phytohormones, which plays an essential role in the response to drought stress in plants. Both exogenous and endogenous increasing ABA concentrations could enhance plant drought tolerance [2,72,73]. Based on the extensive physiological effects of ABA, the role of ABA in improving plant drought tolerance is also multi-layered. On the physical level, ABA induces stomatal closure and cuticular wax accumulation in the epidermal layer during plants subjected to drought stress [74]. On the molecular level, various protein kinases, proteases, enzymes for ROS detoxification and solute, ion and water channel proteins, MAPK cascade, and cross-talk between ABA and JA, GA, collectively regulate plant drought tolerance [75]. Therefore, during prolonged drought, ABA may activate more responses, including enhancing the secondary metabolites biosynthesis, altering starch and sucrose metabolism pathways, etc., to eliminate the damage from drought stress in plants.

## 5. Conclusions

Although large-scale lncRNAs were identified to be involved in abiotic stress, it is still a challenge to uncover the function and mechanism of lncRNAs, and the roles of lncRNAs in ginseng-responsive ABA modulated drought stress remain to be further clarified. Collectively, we identified 17,478 lncRNAs and 3106 new mRNAs by high-throughput illumine sequencing in ginseng. Compared with drought and drought + ABA, the DEGs and DELs target genes mainly enrich in the “biosynthesis of secondary metabolites”, “starch and sucrose metabolism”, and “carbon metabolism” pathways, suggesting that ABA may mediate these pathways to enhance drought tolerance in ginseng. Finally, our results provide new insights into further understanding of lncRNAs function in ginseng drought tolerance. This study also provides a large number of candidate lncRNAs that may serve as markers of drought tolerance genotye screening programs in *P. ginseng*.

## Figures and Tables

**Table 1:** Statistical data of RNA sequencing reads in the 9 samples (libraries) from *Panax ginseng* root treated with drought and drought + ABA treatment. CK1, CK2, CK3, D1, D2, D3, D + A1, D + A2, and D + A3 represent three repetitions of control, drought, and drought + ABA treatment, repectively.

Sample	Raw Reads	Clean Reads	Total Mapped Reads	Unique Mapped Reads	Multiple Mapped Reads	Mapping Percentage (%)
CK1	121040258	119543456	115799005	81367146	34431859	96.87
CK2	92161126	90931870	88183316	57815178	30368138	96.98
CK3	107854860	106614430	103311163	68352727	34958436	96.9
D1	86238756	85001176	80649873	57188222	23461651	94.88
D2	108286158	107061644	103740734	71555652	32185082	96.99
D3	108579332	107195114	103817015	72591330	31225685	96.85
D + A1	118187646	117070450	112842739	82247201	30595538	96.39
D + A2	95475736	93670826	89686391	62471971	27214420	95.75
D + A3	81951742	80954538	76089392	51669000	24420392	93.99

## Data Availability

The complete transcriptome sequencing data have been submitted to Sequence Read Archive under BioProject accession numbers PRJNA1184951 with the BioSample accession numbers SAMN44678709, SAMN44678710, SAMN44678711, SAMN44678712, SAMN44678713, SAMN44678714, SAMN44678715, SAMN44678716, SAMN44678717.

## Supplementary Materials

The following supporting information can be downloaded at: https://www.mdpi.com/article/10.3390/cimb47010005/s1.
